# Neurophysiological Stress Response and Mood Changes Induced by High-Intensity Interval Training: A Pilot Study

**DOI:** 10.3390/ijerph18147320

**Published:** 2021-07-08

**Authors:** Inmaculada C. Martínez-Díaz, Luis Carrasco

**Affiliations:** Department of Physical Education and Sport, University of Seville, E-41013 Seville, Spain; martinezdiaz@us.es

**Keywords:** exercise, HIIT, stress, mood states, cortisol, corticotropin

## Abstract

This pilot study, conducted in advance of a future definitive randomized controlled trial, aimed to investigate the feasibility of using a HIIT-based intervention to induce neurophysiological stress responses that could be associated with possible changes in mood. Twenty-five active male college students with an average age of 21.7 ± 2.1 years, weight 72.6 ± 8.4 kg, height 177 ± 6.1 cm, and BMI: 23.1 ± 1.4 kg/m^2^ took part in this quasi-experimental pilot study in which they were evaluated in two different sessions. In the first session, subjects performed a graded exercise test to determine the cycling power output corresponding to VO_2peak_. The second session consisted of (a) pre-intervention assessment (collection of blood samples for measuring plasma corticotropin and cortisol levels, and application of POMS questionnaire to evaluate mood states); (b) exercise intervention (10 × 1-min of cycling at VO_2peak_ power output); (c) post-intervention assessment, and (d) 30-min post-intervention evaluation. Significant post-exercise increases in corticotropin and cortisol plasma levels were observed whereas mood states decreased significantly at this assessment time-point. However, a significant increase in mood was found 30-min after exercise. Finally, significant relationships between increases in stress hormones concentrations and changes in mood states after intense exercise were observed. In conclusion, our HIIT-based intervention was feasible to deliver and acceptable to participants. A single bout of HIIT induced acute changes in mood states that seems to be associated with hypothalamic-pituitary-adrenal axis activation.

## 1. Introduction

High-intensity interval training (HIIT) consists of short bursts of exercise followed by recovery periods [1]. Thus, HIIT can encompass a considerable range of exercise duration and intensity, either repeated short (<45 s) or long (2–4 min) bouts of rather high- but not maximal-intensity exercise, or short (<10 s, repeated-sprint sequences) or long (>20–30 s, sprint interval session) all-out sprints, interspersed with recovery periods [2]. In any case, there is no doubt that these bouts of intense exercise induce an important physiological stress response which can be observed in different body systems such as muscular, cardiovascular, respiratory, or endocrine ones. In fact, the hypothalamic-pituitary-adrenal (HPA) axis reacts to stress by triggering the secretion of corticotropin release hormone (CRH) from the hypothalamus. CRH stimulates the pituitary to secrete adrenocorticotropin hormone (ACTH) which is the major pituitary hormone regulating adrenal function and, therefore, the release of cortisol, a glucocorticoid regulated by the HPA axis which is secreted by the adrenal gland in response to stress [3,4].

Different studies have been focused on the responses of ACTH to different types of exercise, having demonstrated its reactivity to the most intense ones [5,6]. Furthermore, ACTH responses to two intermittent exercises (1 min of exercise and 30 s of rest) of different intensities (40% and 90% VO_2max_) were evaluated. Plasma ACTH levels were not increased after the less intense exercise; on the contrary, ACTH concentrations increased significantly 10 min after the most intense exercise [7].

As it has been reported, increases in circulating cortisol levels following exercise are related to the extent of stress experienced [8]. Considering that peak cortisol concentrations can be found within 30 min post-exercise and that they could remain elevated up to 2 h, previous studies have found elevated cortisol levels following exhausting exercises [9,10]. More specifically, significant cortisol responses were reported after intense and intermittent exercises [11,12].

On the other hand, there is little information about the effects of vigorous exercise (e.g., HIIT) on mental health and other psychological variables [13]. The few studies that have investigated the acute and long-term effects of interval exercise on psychological outcomes were focused on the perceived exertion, affect, and arousal [14,15]. Several of them reported that HIIT might contribute to feelings of incompetence, failure, and lower self-esteem in untrained subjects, which may undermine participants’ motivation to engage in exercise and sports activities [16]. Several others have investigated the effects of HIIT on affective and enjoyment responses, two key mediator factors for exercise programs adherence, but they have reported contradictory data [17]. However, it seems necessary to link the psychophysiological stress induced by HIIT with changes in mood and behavior. Thus, although some studies have been performed to evaluate the relationships between stress biomarkers and mood states in athletes during training periods suggesting an association between elevated cortisol and dysfunctional mood states [18], nevertheless, there are also contradictory results [19]. Moreover, there is a lack of information about the HIIT-induced cortisol responses and their possible relationships with transient changes in mood states.

Taking into account all above mentioned, we performed this pilot study to address whether a definitive RCT was feasible with regard to (i) assess the impact of HIIT-based intervention on neurophysiological stress response, (ii) determine the subjects’ acceptability to specific HIIT, (iii) estimate possible changes on mood induced by HIIT intervention, and (iv) discover a possible connection between stress hormone response and mood variations after intense exercise.

Thus, the primary objectives of this study were as follows:

A. To assess how many subjects accepted the invitation to participate in this research and meet the inclusion criteria.

B. To determine whether the eligibility criteria for participants were too open or too restrictive by estimating feasible eligibility and recruitment rate.

C. To verify the impact of HIIT-based intervention on neurophysiological stress response and to assess the residual effects on main outcomes. 

D. To investigate the participants’ acceptability to a specific HIIT bout in terms of compliance to this intervention.

E. To assess the acceptability of the outcome measures as methods to measure efficacy of the intervention within a definitive trial.

The secondary objectives of this study were as follows:

a. To evaluate the feasibility of blood sampling from participants’ antecubital vein in pre-, post-, and recovery period after HIIT intervention.

b. To investigate the applicability of POMS questionnaire in pre-, post-, and recovery period after HIIT intervention.

c. To measure precisely key outcome variables (circulating levels of stress hormones, mood states) and to calculate 95% confidence intervals for the difference between assessment time-points.

d. To analyze the association between stress hormone responses and mood changes, from which the definitive RCT could be designed.

## 2. Materials and Methods

### 2.1. Participants

Participants were students at the local Faculty of Educational Sciences, and they were recruited through announcements on social media and academic web sites. Interested participants then registered their interest with the researchers by telephone or email. This was followed up with a screening visit (health history assessment) with the researchers.

Since this was a pilot study, a sample size calculation was not performed. The researchers aimed for more than 20 participants since previous studies reported high effect sizes for stress hormones and mood assessments using between 10 to 26 participants [10,20,21].

The inclusion and exclusion criteria were as follow. Inclusion criteria: male with an age between 18 and 25 years; physically active (i.e., >150 min of moderate-intensity exercise per week for greater than 6 months, according to the International Physical Activity Questionnaire [22]); good general health with no additional disease state that could interfere with the study, and no smoking, drinking alcohol, or drugs use. Exclusion criteria: neurocognitive, psychiatric, or neurological disorders diagnosis (according to DSM-5 criteria) within the last 12 months; any cardiovascular, metabolic, immunological, and endocrine diseases (e.g., Addison’s and Cushing’s diseases), or medication intake that could influence the study results.

Nevertheless, applying inclusion and exclusion criteria we recognized that some participants were taking academic exams. This situation would lead them to experience anxiety or stress episodes, insomnia, and other symptoms which could interfere with the outcomes. We included this issue as exclusion criteria.

### 2.2. Experimental Approach

Considering that this pilot study was conducted to gain experience in delivering the intervention, we decided to develop a quasi-experimental approach because this type of research design allowed us to evaluate the use and acceptability of the HIIT-based intervention and to assess biochemical and psychological outcomes despite limited resources that were available. Thus, a one-group pretest/posttest quasi-experimental design was used in this study in which each participant took part in two testing sessions separated by 5 to 7 days. Body composition analysis (bioelectrical impedance; TANITA BC-418MA); and a graded exercise test on a cycle ergometer (Ergoline Ergoselect 200) was performed during the first session. In the second session, after 15-min of sitting rest, venous blood samples were collected, and Profile of Mood States (POMS) questionnaire [23] was administered. Then, participants performed a HIIT protocol comprising ten 1-min work intervals (cycling at pVO_2peak_) with 1-min of passive recovery in-between. Finally, blood samples were drawn, and POMS questionnaire was filled by participants immediately and 30-min post-HIIT. Within 24 h before testing sessions, participants were instructed to avoid strenuous physical activity, and also to abstain from food (overnight fasting), caffeine, cacao, and alcohol 12 h before testing.

This pilot study was approved by a local University Ethics Committee before the experiment was started (5 February 2017) and performed in accordance with the 1964 Declaration of Helsinki and its later amendments. Accordingly, all participants gave written consent after receiving information regarding the nature and purpose of the study.

#### 2.2.1. Session 1: Graded Exercise Test

Participants performed a maximal incremental exercise test to determine peak VO_2_ (VO_2peak_) on the cycle ergometer. After 5 min of cycling at 50 W, the workload was increased by 25 W per min until exhaustion (cadence: 70 rpm). Breath-by-breath pulmonary gas-exchange data were collected (CPX, MedGraphics, MN, USA) and heart rate (HR) was continuously measured by telemetry (X-Scribe, Mortara, Milwaukee, WI, USA). VO_2peak_ was determined as the highest 20 s mean value reached before exhaustion, defined as the presence of two or more of the following criteria: (1) a maximal rating of perceived exertion (RPE): 19–20 points of 6–20 Borg’s scale [24]; (2) a respiratory exchange ratio above 1.10; (3) a HR above 90% of the age-predicted maximum, and (4) a decrease of cadence below 60 rpm for greater than 5 s. The cycling power output at which participants achieve their VO_2_ peak (pVO_2peak_) was registered for the HIIT intervention (Table 1).

#### 2.2.2. Session 2: HIIT Intervention 

*HR and RPE monitoring.* HR was continuously monitored during HIIT by a HR monitor (Polar RCX5; Polar Electro Ltd., Kempele, Finland). Mean (HR_mean_) and maximal HR (HR_max_) were assessed for each cycling interval. Moreover, the Borg’s 6–20 scale [24] was used to evaluate how participants rated their exertion at the end of each cycling interval to assess their ratings of perceived exertion immediately after each cycling bout.

*Biochemical analyses.* 5-mL blood samples were collected 5 min before, immediately after, and 30-min post HIIT. Plasma ACTH levels were measured using ELISA techniques (AbnovaTM KA3382; Tapei City, Taiwan), with a detection range from 7.9–66.1 pg/mL (no cross-reactivity detectable; inter and intra-assay CV: 5.8 and 3.1%, respectively). On the other hand, plasma cortisol concentrations were also analyzed by ELISA (AbnovaTM KA3382; Tapei City, Taiwan), with a detection range from 30–230 ng/mL, and cross-reactivity with prednisolone (5.1%), corticosterone (0.3%), and progesterone-estradiol (<0.1%). The inter and intra-assay CV were 8.6 % and 6.2%, respectively. All samples were analyzed in duplicate.

*Mood States assessment.* To assess mood, the subjects completed the standard version of POMS [23] at pre-, post-, and 30-min post-exercise. The POMS questionnaire is a 65-item self-administered rating scale which measures six dimensions of mood: depression-dejection (D-D), fatigue-inertia (F-I), tension-anxiety (T-A), confusion-bewilderment (C-B), vigor-activity (V-A), and anger-hostility (A-H). Moreover, POMS Score Index (iPOMS) was calculated using the equation proposed by Fontani et al. [25]:

iPOMS = vigor/((tension + depression + anger + fatigue + confusion)/5)(1)

Nevertheless, according to the objectives of this pilot study, the following clarifications on measures must be considered: (a) the screening visit with the researchers was used to determine how many subjects accepted the invitation to participate in this research and meet the inclusion criteria; (b) percentage of subjects who meet the inclusion criteria was used to determine whether the eligibility criteria for participants were too open or too restrictive by estimating feasible eligibility and recruitment rate; (c) individual rates of perceived exertion were collected to check the impact of HIIT-based intervention on neurophysiological stress response; (d) the acceptability to a specific HIIT intervention was assessed by percentage of subjects who were not able to complete the entire HIIT bout; (e) the administration of a computerized version of POMS and duplicate analyses on ACTH and cortisol plasma levels were used to check the acceptability of the outcome measures as methods to measure effects of the intervention within a definitive trial.

On the other hand, once this pilot study had commenced, we recognized that participants could be experiencing acute psychosocial stress (social evaluation threat) or blood sampling stress in one or both testing sessions. We therefore amended the protocol evaluating participants’ state anxiety levels at the beginning of the testing sessions (State-Trait Anxiety Inventory, STAI) [26] and also by using distraction methods (distraction cards) during blood drawn in those participants who reported needle fear.

### 2.3. Statistical Analysis 

Means and standard deviations (sd) were calculated for all variables. To check normality, Shapiro-Wilk test was conducted. Considering that data were not normally distributed, Friedman Test and Wilcoxon Signed Rank Test were used for intragroup comparisons (mean difference with 95% confidence intervals were also indicated). Moreover, effect size (ES) was calculated using *r*-value (*r* = Z/√N) [27] and was interpreted as trivial when *r* < 0.1, small: *r* = 0.1–0.3, medium: *r* = 0.3–0.5, and large: *r* > 0.5. Bivariate correlations based on both individual raw data and delta percentages were also performed using the Spearman’s *rho*. For all tests, a *p*-value < 0.05 was considered statistically significant.

## 3. Results

### 3.1. Sample Selection

Recruitment began 19 March 2018 and closed on 9 April 2018. The final screening visit was on 4 May 2018. A total of 78 students were initially screened and 25 (32%) were finally included (33 subjects did not meet the following inclusion criteria: daily consumption of alcohol, tobacco, or other drugs, and cardiomyopathy and obstructive respiratory diseases; in addition, 20 subjects were excluded because they were taking academic exams at that moment). The participants’ characteristics are summarized in Table 1.

### 3.2. Graded Exercise Test 

Table 1 shows the participants’ characteristics and performance variables measured during the graded exercise test. Mean VO_2peak_ was 45.3 ± 9.3 mL/kg/min and mean power output at this point (pVO_2peak_) was 275.0 ± 48.9 W. 

### 3.3. HIIT Session: Cardiovascular and RPE Responses

In session 2, all participants were able to complete the entire HIIT bout. Moreover, the state-anxiety scores (STAI) measured prior to HIIT were 17.50 ± 2.39, indicating low anxiety levels.

**Figure 1:**
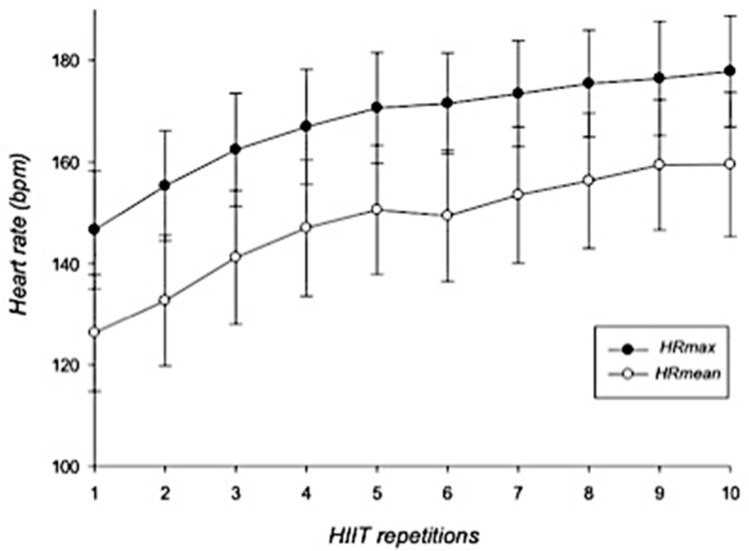
Dynamics of maximal and mean heart rates during HIIT intervention. Error bars represent standard deviations. HR_max_ = maximal heart rate; HR_mean_ = mean heart rate; bpm = beats per minute; HIIT = high-intensity interval training. *n* = 25.

**Figure 2:**
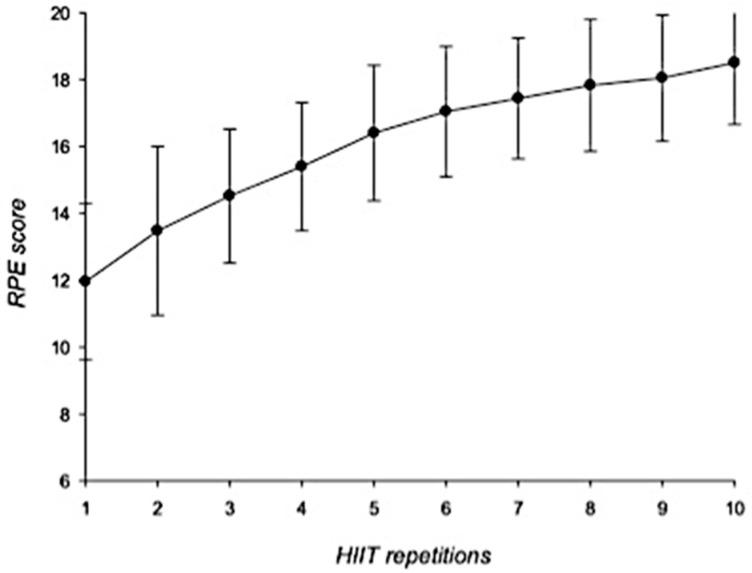
RPE scores during HIIT intervention. Error bars represent standard deviations. RPE = rates of perceived exertion; HIIT = high-intensity interval training. *n* = 25.

In general, HR showed a gradual increase during the HIIT-based intervention. HR_max_ increased from 146.6 ± 11.6 bpm to 177.8 ± 10.9 bpm whereas HR_mean_ increased from 126.3 ± 11.5 bpm to 159.5 ± 14.2 bpm (Figure 1). RPE scores increased from 11.9 ± 2.3 points (first repetition) to 18.5 ± 1.8 points at the last interval (Figure 2).

### 3.4. Plasma ACTH and Cortisol Levels

**Figure 3:**
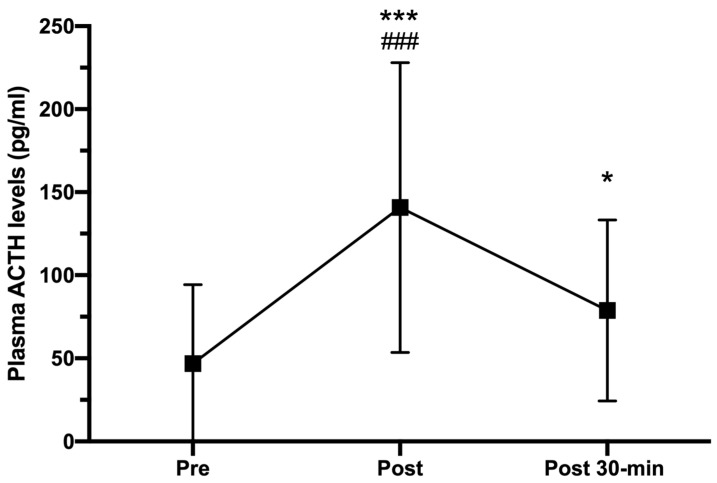
Plasma ACTH concentrations (pg/mL) measured pre-, immediately after (post), and 30-min post HIIT (post 30-min). Error bars represent standard deviations. * *p* < 0.05 and *** *p* < 0.001 compared to pre-HIIT; ### *p* < 0.001 compared to post 30-min. *n* = 25.

As can be seen in Figure 3, plasma ACTH levels showed a significant increase from pre- to post-exercise situation, reaching peak values of 140.71 ± 107.26 pg/mL (CI 95%: −140.57 to −45.51; *p* < 0.001; r = 0.764). However, ACTH concentrations measured 30 min post-HIIT dropped significantly (78.79 ± 64.47 pg/mL; CI 95%: 17.91 to 88.21; *p* = 0.001, r = 0.666), but they were still higher than those observed in the pre-HIIT evaluation (46.82 ± 47.48 pg/mL; CI 95%: −62.17 to −0.12; *p* < 0.05, r = 0.418). 

**Figure 4:**
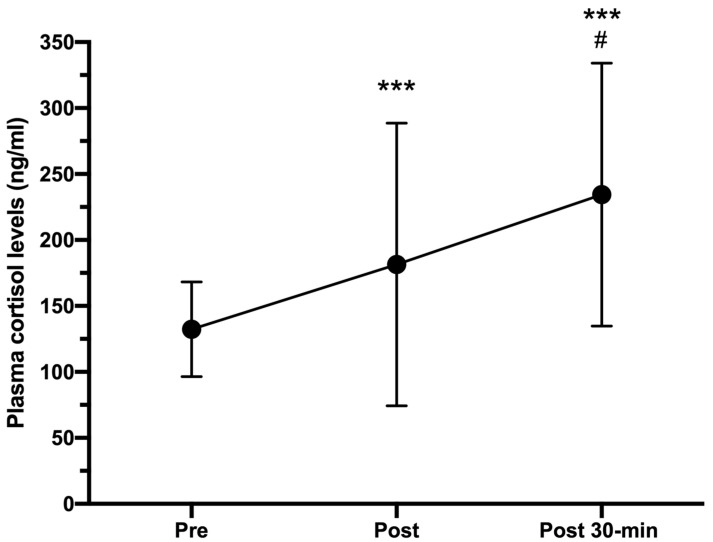
Plasma cortisol concentrations (ng/mL) measured pre-, immediately after (post), and 30-min post HIIT (post 30-min). Error bars represent standard deviations. *** *p* < 0.001 compared to pre-HIIT values; # *p* < 0.05 compared to post-HIIT values. *n* = 25.

Figure 4 shows how plasma cortisol levels progressively increased from pre-HIIT (132.33 ± 35.88 ng/mL) to post-HIIT (181.41 ± 107.26 ng/mL; CI 95%: −79.44 to −29.61; *p* < 0.05, r = 0.716), reaching their highest levels 30 min post-HIIT (234.45 ± 109.63 ng/mL; CI 95%: −144.81 to −59.57; *p* < 0.001, r = 0.828).

### 3.5. Exercise-Induced Mood Changes

**Figure 5:**
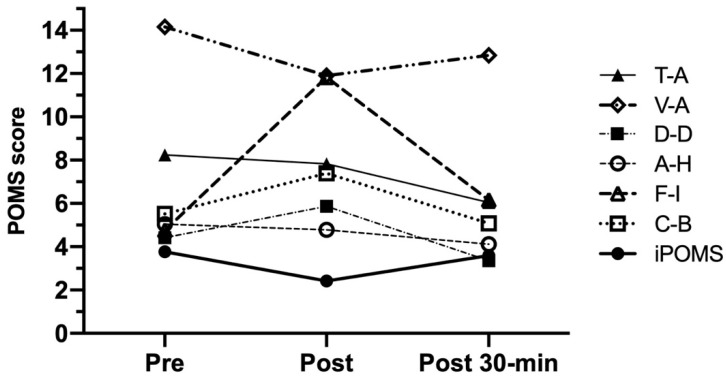
POMS scores measured at the assessment time-points (session 2). Data are presented as mean (*n* = 25). T-A = tension-anxiety; D-D = depression-dejection; A-H = anger-hostility; V-A = vigor-activity; F-I = fatigue-inertia; C-B = confusion-bewilderment; iPOMS = Profile of Mood States Index.

Figure 5 shows the results of POMS dimensions measured at the assessment time-points. T-A and D-D showed a similar trend since they decreased significantly from pre-HIIT to 30-min post-HIIT (CI 95%: 0.553 to 3.847, *p* = 0.036 and CI 95%: 0.333 to 2.787, *p* = 0.015, respectively). Unlike those dimensions, F-I and C-B increased significantly from pre-HIIT to post-HIIT (CI 95%: −8.624 to −4.256, *p* < 0.001 and CI 95%: −2.374 to 0.694, *p* = 0.030, respectively) but they decreased from post-exercise to 30-min post-exercise (CI 95%: 3.137 to 7.559, *p* < 0.001 and CI 95%: 0.402 to 3.946, *p* = 0.021, respectively). However, both A-H and V-A did not show significant changes throughout the three assessment time-points, although V-A scores measured at post-HIIT evaluation were the lowest. 

On the other hand, and as it can be also observed in Figure 5, a significant iPOMS decrease was measured after HIIT (3.77 ± 2.99 and 2.42 ± 2.21 points for pre- and post-HIIT evaluation; CI 95%: 0.58 to 1.96; *p* = 0.002) showing a possible detrimental effect of intense exercise on mood. Although this effect could be explained by a slight decrease in V-A accompanied by significant increases in F-I and C-B observed in post-HIIT assessment time-point, the possibility of a harm effect of evident exercise-induced F-I increases on iPOMS scores must be taken into account for the future RCT study. This is especially significant when other POMS dimensions such as T-A, D-D, and A-H do not seem to be sensitive to high-intensity exercise.

Lastly, and considering the decreases in T-A, D-D, F-I, and C-B scores, iPOMS increased significantly after 30 min of recovery (CI 95%: −2.17 to −0.27; *p* = 0.009), reaching pre-exercise values. 

### 3.6. Relationships between ACTH, Cortisol and Mood States

Using raw individual data, ACTH and cortisol plasma levels were negatively and significatively associated with V-A in pre-exercise situation (Rho = −0.436, *p* = 0.030, and Rho = −0.481, *p* = 0.015, respectively). In addition, ACTH and cortisol plasma levels were positively and significatively correlated with F-I (Rho = 0.620, *p* = 0.002, and Rho = 0.447, *p* = 0.032, respectively) and negatively and significatively with iPOMS (Rho = −0.482, *p* = 0.020, and Rho = −0.467, *p* = 0.025, respectively) in post-exercise situation. Moreover, ACTH showed a positive and significant relationship with C-B (Rho = 0.438, *p* = 0.037) at the same assessment time-point. Finally, a positive and significant correlation was observed between cortisol and C-B measured 30 min post-HIIT (Rho = 0.504; *p* = 0.001). On the other hand, using individual delta percentage of the difference between the assessment time-points, pre-post HIIT ACTH increases were inversely correlated with V-A decreases (Rho = −0.476, *p* = 0.022). 

## 4. Discussion

One of the goals of this pilot study was to investigate the feasibility of using a HIIT-based intervention to induce neurophysiological stress responses that could be associated to possible changes in mood. Considering that all participants were able to complete the entire HIIT bout, the feasibility of our intervention can be stated. The intensity of the HIIT protocol used in this pilot study was set by the cycling power output at VO_2peak_, a physiological variable linked to exercise capacity. Thus, our protocol induced a remarkable increase in both HR and RPE, reaching values near to the maximum. 

The ACTH response to HIIT-related stress was robust. As expected, the values found just at the end of the HIIT bout were significantly higher than those measured before exercise, reaching a percentage increase of up to 200%. Nevertheless, these values significantly decreased 30 min after exercise, reaching levels which were higher than those found before exercise (Figure 3). These results are in line with those previously obtained by other authors when analyzing ACTH responses after intense or exhausting exercises [6,28,29], although the magnitude of the increase was lesser than that observed in previous studies, which found increases of 550% after exhausting exercise on a cycle ergometer [30]. The use of different populations (males, females, or both; trained, recreational or sedentary subjects), age thresholds (young adults, adults, middle aged, or aged), and exercise protocols (intermittent or continuous; vigorous or exhausting; cycling, rowing, or running) could explain the differences in the magnitude of ACTH responses. For example, whereas Schulz et al. [6] evaluated 23 male trained rowers performing a continuous rowing exercise for 9 min at moderate-vigorous intensity, Van der Pompe et al. [28] analyzed 13 postmenopausal women undergoing an incremental exercise on cycle ergometer. Only McMorris et al. [29] and Marquet et al. [30] used healthy young males as participants; however, both studies were focused on ACTH responses to continuous exercises. Moreover, the inter-subject variability in ACTH secretion could play a key role in this respect. Although the CI of differences between pre- and post-exercise assessments were not indicated in these previous studies, we calculated them using the data from Schulz at al. [6]. Thus, the 95% CI range was estimated to be −49.21 to −24.77, much shorter than our CI for ACTH differences (−140.57 to −45.51). On the other hand, and although effect sizes were not reported, the remarkable ACTH responses to exercise observed in the mentioned studies could lead us to estimate large effect sizes, which are consistent with our findings.

Furthermore, it is not surprising that ACTH concentrations decreased 30 min after exercise since the half-life of this hormone in circulation is estimated at 22 min [31]. This trend coincides to that reported previously by other authors, who observed how ACTH plasma levels increased significantly after HIIT session and then decreased remarkably after 10 min of recovery [5,32]. Therefore, and in order to assess the ACTH time course, we can state the feasibility of evaluating ACTH plasma levels up to 30 min during recovery period.

Considering the dynamics of ATCH reported above, and the results obtained in previous studies [11,12,33,34], exercise-induced cortisol responses were to be expected. The magnitude of cortisol responses in our study (37% immediately after HIIT and 77% 30 min post-HIIT) was very similar to those observed in previous studies which reported increases of 31–300% in plasma levels of cortisol that were almost sustained to 1 h post-exercise [35,36]. One more time, differences in samples and exercise protocols used in these studies could explain the wide range of cortisol response magnitude. Additionally (and unfortunately), no data regarding CI and effect sizes are included in these manuscripts so it is difficult to discuss about both the potential inter-individual variability in cortisol responses and the magnitude of different exercise-based interventions, especially those based on HIIT protocols. Nevertheless, our data and those reported by Kujach et al. [35] lead us to consider large effect sizes when definitive RCT is conducted.

On the other hand, and considering that a large number of studies have utilized the POMS questionnaire to evaluate the acute effect of exercise on mood, it is easy to determine what areas of mood are the most positively influenced [37,38]. However, high-intensity exercise has been associated with few desirable changes in mood [38]. In fact, the results of our study showed a significant decrease of iPOMS after HIIT (both fatigue and confusion were significantly increased whereas vigor was slightly decreased) which is in line with the results reported in a recent study [39]. 

As it has been previously reported, affective responses decline as exercise intensity increases beyond the anaerobic threshold [40]. Moreover, several authors have stated that such responses could be influenced by two factors related to HIIT characteristics: the duration of exercise period and the exercise:recovery ratio [17]. Thus, HIIT based on longer exercise periods and imbalanced ratios (e.g., 1:0.5) have resulted in lower affective responses [41,42]. Nonetheless, our results contradict, in part, these hypotheses since we found a decrease in iPOMS after a single bout of HIIT, which was performed using a balanced ratio (1:1).

According with previous studies [43,44] and as we also expected, the 30-min recovery period after HIIT induced decreases in negative mood dimensions of POMS, increasing, consequently, the iPOMS score until reaching pre-exercise levels. Although our pilot study was not mainly focused on the effects of such recovery period on subjects’ ability to recover themselves after intense exercise, data regarding vigor, but especially fatigue, after exercise could serve as indicators of psychophysiological recovery status. Future studies should examine the feasibility of using these POMS dimensions to determine the efficacy of different recovery strategies after high-intensity exercise.

Nevertheless, one of the main findings of our pilot study was that the deleterious effect of HIIT on mood was sustained by the relationships observed between stress hormones (ACTH and cortisol) and POMS dimensions such as fatigue and confusion after intense exercise. To our knowledge, there are few studies that have examined these relationships; nevertheless, our results seem to be consistent with some of them [45,46], although these promising findings need to be confirmed in the definitive RCT.

Lastly, there are some limitations in our study that should be mentioned. As it was previously indicated, the pretest/posttest quasi-experimental design used here has methodological weaknesses that do not allow properly addressing causality. Nevertheless, this pilot study was conducted according to the CONSORT 2010 Statement (extension to randomized pilot and feasibility trials) [47] to gain experience in both delivering the intervention and assessing biochemical and psychological outcomes despite limited resources that were available at that time. Moreover, this pilot study allowed us to identify potential biases that could affect the treatment effect in the future definitive RCT. Some of them are recruitment-related biases; the recruitment period would be crucial since in this pilot study 20 subjects were excluded because they were taking academic exams (and they might be suffering for anxiety or stress). This could have implications for progression from pilot to future definitive trial. In fact, eligibility of the screened volunteers was lower than expected, indicating that both recruitment time and locations should be improved.

Moreover, stress associated with social evaluation threat or with blood sampling could affect significantly the outcomes of future RCT. To avoid these intervention biases it will be necessary (a) to evaluate the state-anxiety of subjects prior to each testing sessions, and (b) to apply distraction methods (e.g., distraction cards) during blood drawn in those participants who reported needle fear. Although in the present pilot study all reported state-anxiety levels were below the population median, it could be necessary to exclude from the analysis those participants who show high-levels of anxiety. Finally, measuring biases might be considered if iPOMS is used as mood states-related outcome. Although negative mood dimensions such as tension-anxiety, depression-dejection, anger-hostility, and confusion-bewilderment do not change after exercise, it would be possible that exercise-induced fatigue leads subjects to accentuate the fatigue-inertia dimension score, modulating in consequence the iPOMS score. Thus, if POMS is used, individualized analyses of each dimension are recommended. Additionally, the concurrent use of scales for estimating affective and enjoyment responses would allow us to obtain a more comprehensive understanding the potential role of high-intensity exercise in mood, affective feelings, and other related emotional states.

Our data reflect the activities of only one pilot trial; in fact, the sample characteristics may have also limited the generality of our findings. According to the characteristics of our HIIT-based intervention, we only used physically active male college volunteers, so the results are not generalizable to females or sedentary populations. However, we hope that the methods may serve as a template for analyzing other pilot studies with similar or different designs in other exercise-related settings.

## 5. Conclusions

According to the results obtained in this study, we can state that a single bout of HIIT increases the feelings of fatigue and confusion-bewilderment in healthy young males which could affect their mood states. This negative effect seems to be associated with HPA-axis activation and the increase of circulating levels of ACTH and cortisol. Future RCT studies should emphasize the influence of these stress hormones on HIIT mood responses by using specific ACTH or cortisol blockers and to evaluate the modulating effect of subjects’ exercise-related expectations, since in combination with the psychophysiological mechanisms involved in intense exercise, they could jointly determine the perceived and experienced psychological effects of exercise.

## Figures and Tables

**Table 1:** General characteristics of subjects and results from the graded exercise test (session 1).

	Mean	sd
Subjects’ characteristics (*n* = 25)		
Age (yr)	21.7	2.1
Height (cm)	177	6.1
Weight (Kg)	72.6	8.4
BMI (Kg/m^2^)	23.1	1.4
Body fat (%)	13.4	3.6
IPAQ-total score (MET/min/wk)	5877.6	1668.2
Graded Exercise Test		
Time to exhaustion (min)	13.6	1.8
HR_max_ (bpm)	181.5	8.4
VO_2peak_ (mL/kg/min)	45.3	9.3
pVO_2peak_ (W)	275.0	48.9

BMI = body mass index; HR_max_ = maximal heart rate; VO_2peak_ = peak oxygen consumption; pVO_2peak_ = cycling power corresponding; sd = standard deviation.

## Data Availability

The data presented in this study are available on request from the corresponding author.
